# Children’s social evaluation toward prestige-based and dominance-based powerholders

**DOI:** 10.1186/s13104-022-06072-6

**Published:** 2022-05-15

**Authors:** Masahiro Amakusa, Xianwei Meng, Yasuhiro Kanakogi

**Affiliations:** Graduate School of Human Sciences, Osaka University, 1-2, Yamadaoka, Suita, Osaka 565-0871 Japan

**Keywords:** Dominance, Prestige, Social hierarchy, Children, Social evaluation

## Abstract

**Objective:**

Social scientists have suggested two typical ways of acquiring social power: dominance approach (gaining social power by applying violence, coercion, threat, and punishment) and prestige approach (gaining admiration and liking by demonstrating competence and sharing experience and knowledge). However, little is known about how people recognize and evaluate the differentiated process of the approaches, and even less about the early development of these processes. In the current study, 5–6-year old children heard stories about pairs comprising a dominance-based and a prestige-based powerholder, chose one of the powerholders as their friend and leader, and predicted which powerholder will gain the contested resources.

**Results:**

Compared to a dominance-based powerholder, children were more likely to choose a prestige-based powerholder as a friend and leader in different situations. Moreover, children predicted that prestige-based powerholders, and not dominance-based powerholders, would gain contested resources. These findings suggest that since childhood, human beings tend to be biased to not only judge prestige-based aspects as socially preferable, but also endorse the prestige-based powerholders’ priority to possess valuable resources, which subsequently strengthens their high social status. These early childhood preferences can be instrumental in providing more harmonious environments for children in educational and daily contexts.

**Supplementary Information:**

The online version contains supplementary material available at 10.1186/s13104-022-06072-6.

## Introduction

Hierarchical structure is a universal aspect of human groups; higher-ranking individuals typically hold social power to asymmetrically control fitness-relevant resources (e.g., food, mates) and influence group decisions [1–3]. To understand the psychological foundations of hierarchy establishment processes among individuals, various theoretical and empirical notions have been put forward by scholars across scientific disciplines, such as anthropology [4] and evolutionary biology [5]. A well-established view suggests that social power can be acquired through two ways: the dominance approach and the prestige approach [6]. Specifically, dominance depicts a fear-based approach to gain social power by applying violence, coercion, threat, and punishment to (potential) subordinates, whereas prestige depicts a respect-based approach of gaining admiration and liking from others by demonstrating competence and sharing experience and knowledge. Although studies have shown that human adults’ attitudes towards powerholders depend on the approaches through which they acquire power [7, 8], little is known about the evaluation in early stages of human development.

It has been known that preschoolers use various cues (e.g., upright posture; [9]) to infer social power (e.g., “to be the boss”; [10]) and that the explicit distinction between dominance and prestige emerges at 5 years old [11]. With regard to social evaluation towards powerholders, Cheng et al. presented children with a dominance-based powerholder (who obtains power by demonstrating physical strength and giving orders) and a prestige-based powerholder (who is highly skilled in particular activities and is elected as the boss), and found that 6-year-old children are more likely to choose the prestige-based powerholder as their friend and leader [12]. This suggests that, compared to the dominance-based powerholder, children are more likely to have an affinity with, and respect for, the prestige-based powerholder.

However, it is unclear which type of powerholders children think should have a greater likelihood of gaining valuable resources. Studies in evolutionary biology have suggested that possessing valuable resources is crucial for powerholders to maintain high social status [13, 14]. Therefore, understanding children’s expectations would not only cultivate a better understanding of how they recognize and evaluate power obtaining processes but also provide cues to understand how human’s hierarchical society is established. In addition, the context where the powerholders were introduced in the previous study was limited; both the dominant and the prestige individuals were described in one story (situation). Therefore, it was unclear whether children’s evaluation of the powerholders could extend to other situations, which would help draw a more complete picture of their perceptions regarding the prestige and the dominance approaches.

To fill the gaps in the literature described above, the current study conceptually replicated and extended Cheng et al.’s study with the following modifications and aims [12]. First, we showed children the situation where prestige-based and dominance-based powerholders competed for valuable resources, and asked them to predict which powerholder will succeed in gaining the resources. Second, we presented them with three different contexts where dominant and prestigious powerholders were introduced.

In adult society, individuals’ ability and willingness to help others (i.e., generate benefits for others; prestige approach) are strong predictors of status in the minds of others, but individuals’ ability and willingness to impose their will on others (i.e., inflict cost on others; dominance approach) do not reliably increase status in the minds of others [15]. Therefore, we expected that children are more likely to predict that the prestige-based powerholder will gain valuable resources. Also, based on Cheng et al.’s findings, we hypothesized that, compared to the dominant powerholder, children are more likely to befriend the prestigious powerholder and elect them as the leader [12].

## Main text

### Methods

#### Participants

Thirty-nine 5–6-year-old children (*M* = 72.5 months, range = 66–79 months, *SD* = 3.6 months; 16 girls) participated in the experiment. They were recruited from and tested in their kindergarten, in Osaka, Japan. Written informed consent was obtained from children’s caregivers. Three participants were excluded could not complete or had incorrect responses to the memory checking questions. A power analysis indicated that at least 39 participants will be required to detect potential effects using a one-sample Wilcoxon signed-rank test with a medium effect size (*d* = 0.5), significance *α* = 0.05, and power 1−*β* = 0.80 [16]. The research reported in this manuscript was approved by the Behavioral Research Ethics Committee of the Osaka University School of Human Sciences (HB021-066).

#### Set-up

The experiment was conducted in a quiet room in the kindergarten. Each child was seated facing the experimenter and was presented with three picture-stories. They were asked to answer a series of questions regarding the stories. All utterances were recorded by a digital voice recorder.

#### Task

Each story presented specific situation (context) in which two powerholders gain social power from their subordinates through different processes: a dominance-based powerholder gains social power by applying threat to subordinates, while a prestige-based powerholder gains social power by demonstrating competence and sharing knowledge with subordinates (Additional file 1: Figure S1). The “Original story” was identical to that of Cheng et al. [12]. The dominance-based powerholder claims his strong body, threatens peers so that in their play, they must use the powerholder’s favorite toys (not choose others’ favorite toys), whereas the prestige-based powerholder is invited by peers to play together, and is asked to choose a toy for the play because he has won a drawing contest and is respected by the peers. Other stories were created based on the original story. In the “King story,” the dominance-based powerholder (a scary king) violently scolds those who do evil, to maintain peace in the country, whereas the prestige-based powerholder (a kind king) shares knowledge with people to maintain peace in the country. In the “Jump rope story,” the dominance-based powerholder is physically strong, good at fighting, and peers listen to him because they are afraid of him; the prestige-based powerholder is good at jump rope and kindly teaches the peers to jump rope, hence, they listen to him.

The order of the stories was counterbalanced across participants. For each participant, the powerholder who was introduced first among the three stories was fixed as “dominance-based, prestige-based, dominance-based” or “prestige-based, dominance-based, prestige-based.” Sex of all powerholders introduced in the stories was matched to the participant’s sex.

After hearing each story, the experimenter tested whether participants had correctly remembered the powerholders. Specifically, the experimenter showed participants the pictures of the two powerholders and asked questions regarding each of the powerholders: “What is this person like?” (Memory test) If participants could not give appropriate answers (e.g., he is good at soccer), the experimenter repeated the story until they could pass the memory test.^1^ Thereafter, participants answered three target questions and provided justifications for their answers after each question (Table 1).

**Table 1:** Target questions used in the current study

Type of question	Question
*The friend-preference question*	Participants were asked: “Who do you want to be good friends with between these two persons?”
*The leader-preference question*	Participants were asked: “These two persons are claiming to go toward different places. Which person would you want to follow as a leader?” Although studies usually use linguistic expressions (e.g., “boss”, “in charge” [17, 18]; to directly depict boss and leader, our pilot investigation found that Japanese preschoolers do not understand well the meaning of such linguistic expressions. Therefore, we used the leader–follower context, in which children choose a target as the leader to follow. Note that “leader” may function in dimensions more than just “being followed” (e.g., giving orders [18]; but this does not deny that “being followed” is a typical dimension that preschoolers can understand [19])
*The resource-gaining-prediction question*	This question aimed to test which type of powerholders children think should gain a valuable resource [17, 20]. Participants saw a vertical arrangement of two chairs, where the upper chair is bigger and more attractive than the lower chair. The experimenter instructed the participants: “There are two chairs here: one is in a higher position, is bigger, and more attractive than the other. Two persons (the dominance-based powerholder and the prestige-based powerholder) want to sit on the more attractive chair and compete for it. See the following pictures and tell me who should sit on the upper chair?”

#### Scoring and analysis

To determine whether, compared to the dominance-based powerholder, the children were more likely to choose the prestige-based powerholder as friend, leader or owner of the contested resource, we calculated a preference score and tested whether its mean was statistically different from chance (i.e., a score of 1.5). The preference score indicated the child’s total number of choices for the prestige-based powerholder across three stories (range= 0–3). As the data were expected to show non-normal distributions, we used a one-sample Wilcoxon signed-rank test for hypothesis testing. The data for each event type were analyzed separately. We used a p-value of 0.05.

### Results

**Fig. 1:**
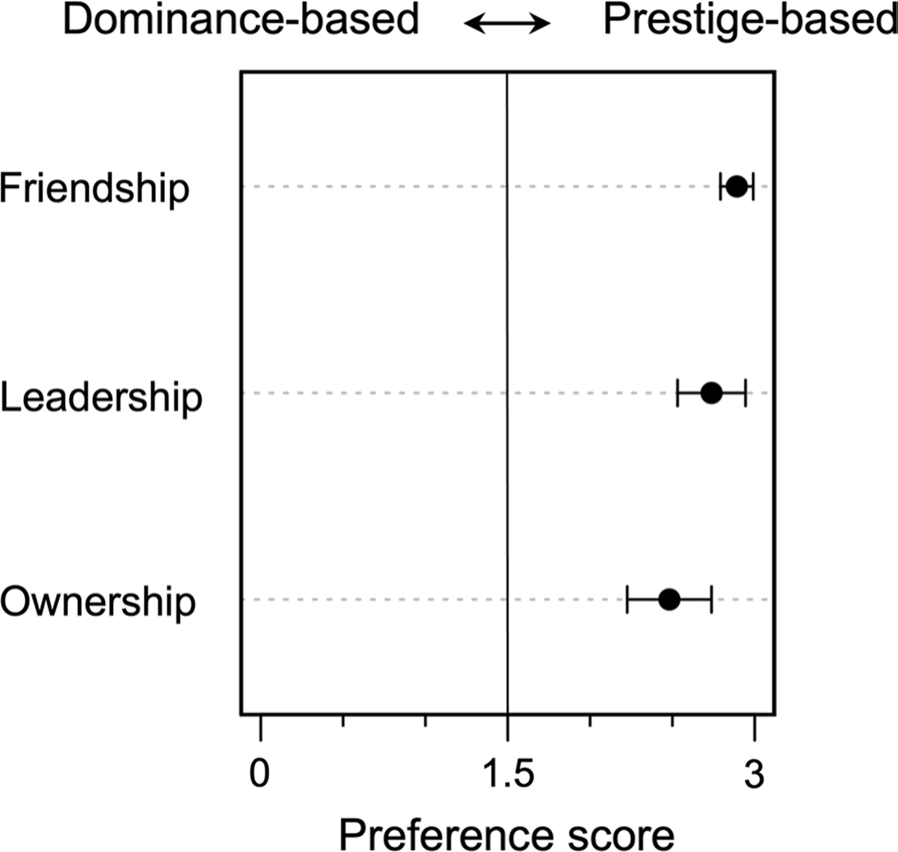
Children’s judgements on all three questions. Black points show the mean of the preference score and the error bar presents 95% confidential intervals

Children’s preference of choosing the prestige-based powerholder (not the dominance-based) was significantly above chance for all the questions. For the friend-preference question, *M* = 2.90, *SD* = 0.307, *V* = 780, *p* < 0.001, and *r*_*rb*_ = 1.000 (Fig. 1). For the leader-preference question, *M* = 2.74, *SD* = 0.637, *V* = 753, *p* < 0.001, and *r*_*rb*_ = 0.932. For the resource-gaining-prediction question, *M* = 2.49, *SD* = 0.790, *V* = 731, *p* < 0.001, and *r*_*rb*_ = 0.874.

Furthermore, we tested whether children’s response was influenced by the type of stories and other uninterested factors (i.e., gender). For response on each question, a generalized linear mixed model was applied with binomial error and logit link functions (1 = choosing prestige-based powerholder, 0 = choosing dominance-based powerholder) and included children’s age (in months), gender, and type of story as fixed factors, and participant ID as random effects. Results were similar across three questions; no factors were found to influence children’ response (*p*s > 0.05).

To explore the psychological process of children’s evaluation regarding the ownership, we investigated the reasons why children predicted the dominance-based or prestige-based powerholder to obtain the contested resource (Results and discussion of justifications of friendship and leadership preferences can be found in the Additional file 1). Results showed that, among 94 judgements of children who predicted that the prestige-based powerholder would obtain the attractive chair, 60 referred to powerholder’s prosocial character (e.g., “He is kind.”), 16 referred to the powerholder’s high competence (e.g., “He is good at drawing pictures.”), nine referred to the powerholder’s knowledge-sharing behavior (e.g., “He teaches others various things.”), and 14 mentioned other reasons (e.g., “I don’t know.”). Among 23 judgements of children who predicted that the dominance-based powerholder would obtain the attractive chair, 13 mentioned the powerholder’s physical strength (e.g., “He looks strong.”), two mentioned the characters (e.g., “He looks angry.”) and eight mentioned other reasons (e.g., “*The prestige-based powerholder* would give way to *the dominance-based powerholder*”).

### Discussion

This study showed that, compared to a dominance-based powerholder, 5–6 year old children are more likely to choose a prestige-based powerholder as a friend and leader in different situations. These findings extend those of Cheng et al. by suggesting that children’s preference toward prestige-based powerholders is not limited to the specific context used in their study, but can be generalized to a wide range of contexts [12].

A study has shown that young children predict resource allocation based on the hierarchical structure between the recipients, which could be inferred through the past interactions of the recipients [17]. The current study first showed that, when both recipients are powerholders, children predicted that prestige-based powerholders, and not dominance-based powerholders, would gain contested resources. Theoretical considerations have shown that possessing valuable resources is crucial for powerholders to maintain high social status [13, 14], and empirical evidence has shown that children infer social power based on resource control [9, 19]. Therefore, the current findings suggest that, compared to the dominance-based powerholders, children are biased to endorse that the prestige-based powerholders should have higher social status. Moreover, the majority of the judgements were made taking into account the prestige-based powerholder’s prosocial character (See the similar evaluation process regarding friendship preference and leadership preference; Additional file 1). This is in line with the phenomenon that human adults attribute higher social status to individuals who show a willingness to generate benefit for others [15].

The current findings might represent a new wave of research aiming to understand how human beings, from early developmental stage, link social power and personal characteristics. Toward more important and broader impacts, the preferences toward prosocial and moral leaders in early childhood, would inspire practical approaches for providing children more harmonious environments in educational and daily contexts (e.g., educational instructions can be more effective if they are given by individuals who have prestige-related characters).

## Limitation

Two limitations which would inspire future investigations in this area must be considered. First, the current findings may be limited to specific cultures (i.e., Japanese culture). A previous study has demonstrated cross-cultural differences in the valuing of dominance in childhood [21]. For instance, compared to French preschoolers, Japanese preschoolers were more likely to believe a subordinate than a dominant individual. Therefore, cross-cultural investigations should be conducted to test the generalization of the current findings. Second, the developmental origin and trajectory of differential evaluation toward prestige-based and dominance-based powerholders is not clear. Hence, future studies should test the responses from other developmental stages, incorporating the methodologies of studies with infants and adults [15, 22–25].

## Supplementary Information

**Additional file 1: Figure S1.** Stimuli used in the study. The numbers indicate the presentation order of the pictures.

## Data Availability

The datasets used and/or analyzed during the current study are available from the corresponding author on reasonable request.

## Abbreviations

M: Mean
SD: Standard deviation
r_rb_: Rank-biserial correlation

## Footnotes

1 The maximum number of repetitions were three. Since three children could still not answer correctly after the story was repeated three times, they were excluded from the final sample.
