# Supplementary material for: Children’s social evaluation toward prestige-based and dominance-based powerholders
Source: BMC Res Notes. 2022 May 15;15:180. doi: 10.1186/s13104-022-06072-6 (PMC9107631; doi:10.1186/s13104-022-06072-6)
Supplement: Supplementary file 1 — Additional file 1: Figure S1. Stimuli used in the study. The numbers indicate the presentation order of the pictures. [file 13104_2022_6072_MOESM1_ESM.docx]

Additional file

**Children’s social evaluation toward prestige-based and dominance-based powerholders**

Masahiro Amakusa^1^, Xianwei Meng^1*^, Yasuhiro Kanakogi^1^

^1^Graduate School of Human Sciences, Osaka University, Suita, Japan

*Correspondence concerning this article should be addressed to:

Xianwei Meng,

1-2, Yamadaoka, Suita, Osaka 565-0871, Japan

+81-6-6879-8045

Email: mokeni1211@gmail.com.

**Additional file Figures**

**Figure S1.** Stimuli used in the study. The numbers indicate the presentation order of the pictures.

**Justifications of friendship and leadership preferences**

To explore the psychological process of children’s evaluation regarding friendship and leadership, we investigated the reasons why children chose dominance-based or prestige-based powerholders as good friends or leaders. With regard to the friendship preference, among 112 judgements of children who chose the prestige-based powerholder as a good friend, 68 referred to the powerholder’s prosocial character (e.g., “She is a nice girl.”), 13 referred to the powerholder’s high competence (e.g., “He is good at drawing pictures.”), 13 referred to the powerholder’s knowledge-sharing behavior (e.g., “She teaches others many things.”), and 21 mentioned other reasons (e.g., “I don’t know.”). Among five judgements of children who chose the dominance-based powerholder as a good friend, one mentioned the powerholder’s physical strength (e.g., “He looks strong.”) and four mentioned other reasons (e.g., “I do not know.”).

With regard to the leadership preference, among 107 judgements of children who chose the prestige-based powerholder as a leader, 64 referred to the powerholder’s prosocial character (e.g., “He is kind.”), seven referred to the powerholder’s high competence (e.g., “She is good at jump rope.”), five referred to the powerholder’s knowledge-sharing behavior (e.g., “He teaches others many things.”), and 32 mentioned other reasons (e.g., “I don’t know.”). Among ten judgements of children who chose the dominance-based powerholder as leader, four mentioned the powerholder’s physical strength (e.g., “She looks strong.”), two mentioned powerholder’s character (e.g., “He is a strict boy.”), and four mentioned other reasons (e.g., “I do not know.”).

Descriptive results show that the majority of the judgements in which children chose the prestige-based powerholders as friends or leaders were made taking into account the prestige-based powerholders’ prosocial character. This suggests that children are motivated to establish positive relationships with prestige-based powerholders and to follow them as leaders because the prestige-based powerholders are prosocial individuals who show seek to benefit others (1–3). Interestingly, children seem to be more likely to value the cues of such socio-moral properties than competence when evaluating powerholders as friends and leaders. As discussed in the main text, similar evaluation process could be found when children predict the ownership of a contested resource: they value the powerholders’ prosocial character (4). These findings indicate that children’s social evaluation towards powerholders basically depend on the perceived willingness of the powerholders to generate benefit for others (5).

**References**

1. Gurven M, Allen-Arave W, Hill K, Hurtado M. “It’s a wonderful life”: signaling generosity among the Ache of Paraguay. Evol Hum Behav. 2000;21(4):263–82.

2. Delton AW, Robertson TE. The social cognition of social foraging: Partner selection by underlying valuation. Evol Hum Behav. 2012;33(6):715–25.

3. Lukaszewski AW, Simmons ZL, Anderson C, Roney JR. The role of physical formidability in human social status allocation. J Pers Soc Psychol. 2016;110(3):385.

4. Thomas AJ, Thomsen L, Lukowski AF, Abramyan M, Sarnecka BW. Toddlers prefer those who win but not when they win by force. Nat Hum Behav [Internet]. 2018 Sep 7;2(9):662–9. Available from: http://www.nature.com/articles/s41562-018-0415-3

5. Durkee PK, Lukaszewski AW, Buss DM. Psychological foundations of human status allocation. Proc Natl Acad Sci U S A. 2020;117(35):21235–41.
